# The Influence of Gendered Social Position on Cohort Differences in Smoking Among Older Mexican Adults

**DOI:** 10.1093/geroni/igab046.2274

**Published:** 2021-12-17

**Authors:** Sadaf Milani, Jaqueline Avila, Rebeca Wong

**Affiliations:** 1 University of Texas Medical Branch, Galveston, Texas, United States; 2 Brown University, United States

## Abstract

Disparities throughout the life course, including social position, result in gendered pathways to health, which ultimately result in gender disparities in late life. Using data from the Mexican Health and Aging Study, we explore the concept of gendered social position over the life course (educational achievement, marital history, employment history) and its association with smoking status in old age (current, former, never). We compare two cohorts by gender, those aged 60 to 71 in 2001 (n=4,383) and 60-71 in 2012 (n=5,970), as these cohorts experienced vastly different life courses. Overall, current smoking decreased from 2001 to 2012, but men consistently report higher rates of current and past smoking, compared to women. This presentation will widen the lens of gender to consider the influence of social position established over the life course, on gender differences in smoking, a risk factor for poor health and function in old age.

